# Discrepancies between qualitative and quantitative evaluation of randomised controlled trial results: achieving clarity through mixed methods triangulation

**DOI:** 10.1186/s13012-016-0436-0

**Published:** 2016-05-12

**Authors:** Sarah Tonkin-Crine, Sibyl Anthierens, Kerenza Hood, Lucy Yardley, Jochen W. L. Cals, Nick A. Francis, Samuel Coenen, Alike W. van der Velden, Maciek Godycki-Cwirko, Carl Llor, Chris C. Butler, Theo J. M. Verheij, Herman Goossens, Paul Little, on behalf of the GRACE INTRO/CHAMP consortium

**Affiliations:** 1 Nuffield Department of Primary Care Health Sciences, University of Oxford, Oxford, UK; 2 Department of Primary Care and Interdisciplinary Care, University of Antwerp, Wilrijk, Antwerp, Belgium; 3 South East Wales Trials Unit, Centre for Trials Research, Cardiff University, Cardiff, UK; 4 Academic Unit of Psychology, University of Southampton, Southampton, UK; 5 Department of Family Medicine, CAPHRI School for Public Health and Primary Care, Maastricht University, Maastricht, The Netherlands; 6 Cochrane Institute of Primary Care and Public Health, School of Medicine, Cardiff University, Cardiff, UK; 7 Vaccine and Infectious Disease Institute (VAXINFECTIO), Laboratory of Microbiology, University of Antwerp, Antwerp, Belgium; 8 Julius Center for Health Sciences and Primary Care, University Medical Center Utrecht, Utrecht, The Netherlands; 9 Faculty of Health Sciences, Medical University of Lodz, Lodz, Poland; 10 Primary Healthcare Centre Via Roma, Barcelona, Spain; 11 Primary Care and Population Sciences, University of Southampton, Southampton, UK

**Keywords:** Antibiotic, Respiratory infection, Intervention, Mixed methods, Triangulation, Qualitative

## Abstract

**Background:**

Mixed methods are commonly used in health services research; however, data are not often integrated to explore complementarity of findings. A triangulation protocol is one approach to integrating such data. A retrospective triangulation protocol was carried out on mixed methods data collected as part of a process evaluation of a trial. The multi-country randomised controlled trial found that a web-based training in communication skills (including use of a patient booklet) and the use of a C-reactive protein (CRP) point-of-care test decreased antibiotic prescribing by general practitioners (GPs) for acute cough. The process evaluation investigated GPs’ and patients’ experiences of taking part in the trial.

**Methods:**

Three analysts independently compared findings across four data sets: qualitative data collected view semi-structured interviews with (1) 62 patients and (2) 66 GPs and quantitative data collected via questionnaires with (3) 2886 patients and (4) 346 GPs. Pairwise comparisons were made between data sets and were categorised as agreement, partial agreement, dissonance or silence.

**Results:**

Three instances of dissonance occurred in 39 independent findings. GPs and patients reported different views on the use of a CRP test. GPs felt that the test was useful in convincing patients to accept a no-antibiotic decision, but patient data suggested that this was unnecessary if a full explanation was given. Whilst qualitative data indicated all patients were generally satisfied with their consultation, quantitative data indicated highest levels of satisfaction for those receiving a detailed explanation from their GP with a booklet giving advice on self-care. Both qualitative and quantitative data sets indicated higher patient enablement for those in the communication groups who had received a booklet.

**Conclusions:**

Use of CRP tests does not appear to engage patients or influence illness perceptions and its effect is more centred on changing clinician behaviour. Communication skills and the patient booklet were relevant and useful for all patients and associated with increased patient satisfaction. A triangulation protocol to integrate qualitative and quantitative data can reveal findings that need further interpretation and also highlight areas of dissonance that lead to a deeper insight than separate analyses.

**Electronic supplementary material:**

The online version of this article (doi:10.1186/s13012-016-0436-0) contains supplementary material, which is available to authorized users.

## Background

Mixed methods research, combining qualitative and quantitative methods, is becoming increasingly common in health services research in recognition of the different types of research question each can answer [1]. Whilst mixed methods are frequently used, data analysis is often carried out separately and findings are not integrated between data sets. Various approaches have been identified to integrate data in mixed methods research with a “triangulation protocol” as one example [1]. Here, triangulation refers to the integration of different approaches to gain a more complete picture and provide a “whole which is greater than the sum of the parts” and various types of triangulation exist (detailed below) [1, 2]. A triangulation protocol can also enhance the validity of findings and assess whether data agree (convergence), complement one another (complementarity) or contradict each other (dissonance) [1]. Dissonance, in this respect, does not indicate a failure in the study but can be considered constructive if it leads to new findings or a richer understanding [3].

Some caution is advised when carrying out triangulation because of its potential complexities. One recommendation suggests having researchers with appropriate expertise in qualitative and quantitative methods to ensure data is handled appropriately [2]. The combination of qualitative and quantitative data can also lead to clashes in the philosophical assumptions behind each approach and therefore recommendations have been made for triangulation to be carried out from a pragmatic, or subtle realist, approach [1]. Four types of triangulation have been identified in the literature: (1) methodological, use of more than one research method or data collection technique; (2) data, use of multiple data sources; (3) theoretical, use of multiple theories; and (4) investigator, use of two or more researchers in the analysis [4]. The types of triangulation used in a project should reflect the individual research question. To date, there are few examples of a triangulation protocol in the literature and more work is needed to establish how the technique can contribute to research studies [1, 2].

In the present study, a mixed methods process evaluation was carried out as part of a programme of work investigating the effectiveness of interventions to promote prudent use of antibiotics in general practice. A large, multi-country factorial cluster randomised controlled trial (RCT) examined the effectiveness of two interventions aimed at decreasing antibiotic prescribing for acute cough by general practitioners (GPs) [5]. Use of web-based communication skills training together with an interactive patient booklet and web-based training to use a point-of-care C-reactive protein (CRP) test together with the installation of the test device in the GP practice were evaluated in a 2 × 2 factorial design across six European countries. General practices were cluster randomised to one of four groups: (i) control, (ii) training in communication skills and use of a patient booklet, (iii) training in use of a CRP test and provision of the test device or (iv) both interventions (communication skills and CRP test). The two interventions led to important decreases in antibiotic prescribing with fewer antibiotics being prescribed in groups which received both interventions [5].

The process evaluation of the trial collected both quantitative and qualitative data. Data collection was carried out sequentially, and the original, planned process evaluation reported on separate analyses of the two types of data [6–8]. The current study aimed to follow a triangulation protocol to integrate mixed methods data previously collected in order to see whether such an approach could further inform the findings of the original process evaluation of the trial.

## Methods

### Study design

This study involved carrying out a triangulation protocol to integrate quantitative and qualitative data as part of a process evaluation of an RCT of a complex intervention [9].

### Setting and participants

The trial was carried out in six countries, Belgium, England, the Netherlands, Poland, Spain and Wales, and included 246 general practices. Both qualitative and quantitative data were collected from GPs and patients who had taken part in the trial.

### Intervention materials

Training in both of the interventions was delivered to participating GPs via a web-based programme that was viewed as acceptable and feasible for use by GPs [10]. The web-based programme consisted of three modules; an introduction, communication skills training and CRP training. All GPs in the three intervention groups received the introduction module and either one of the two training modules or both modules. The control group did not receive training.

Patients in intervention groups with communication skills training received a booklet providing information about how to manage a cough without antibiotics. Patients in the intervention groups with CRP test received a CRP test in their consultation if their GP felt this was needed (GPs were instructed to carry out a test if they were considering prescribing antibiotics). Further information about the content of the web-based training, the CRP test and the patient booklet is provided elsewhere [5, 8].

### Data collection

Quantitative data were collected via self-report. All patients and GPs who had taken part in the trial were sent questionnaires. GPs were presented with an online baseline questionnaire prior to taking part and a questionnaire once the trial had been completed. The items asked about GPs’ views on prescribing antibiotics for acute respiratory infections and, the intervention delivery, their views on using the CRP test, patient booklet and communication skills training. The patients were provided with questionnaires when they consented to take part in the trial at the time of consultation. The items asked about patients’ perceptions of antibiotics, whether they had received all the information they wanted and their satisfaction with the consultation. Further details of the quantitative data collected are provided elsewhere [8].

Qualitative data were collected via interviews with patients and GPs who had been in one of the intervention group of the trial (communication, CRP or both). Both patients and GPs were asked about their experiences of taking part in the trial and their views of either the intervention training they had received (GPs) or the consultation they had attended (patients). GPs were invited to take part in an interview once their practice had completed the trial; patients were invited shortly after their initial consultation. Participants were not aware of the outcome of the trial at the time of interview. Interviews followed semi-structured interview guides for each participant group. Further details about recruitment and data collection are provided elsewhere [6, 7].

### Analysis

Data were initially analysed in three separate analyses; quantitative patient and GP data (led by LY), qualitative patient data (led by ST-C) and qualitative GP data (led by SA). The methods used to originally collect and analyse data are reported elsewhere [6–8].

This paper reports the results of a triangulation protocol where data from all four sources (quantitative and qualitative for both patients and GPs) were compared. A triangulation protocol involves the integration of data when all sets of data have already been analysed individually. Four types of triangulation are identified in the literature (methodological, data, theoretical and investigator) and the current study used three of these approaches. These included methodological triangulation, with the use of more than one data collection technique (interviews and questionnaires), data triangulation, with the use of multiple data sources (text and numbers) and investigator triangulation using three analysts [4]. Theoretical triangulation was not applicable for this study as all research activities had been carried out from a realist perspective [2]. Using three types of triangulation incorporated a variety of approaches which strengthened the analysis by taking a more holistic approach to collecting and analysing data and evaluating the trial [1].

The original data, interpretation and reports of all analyses were examined, and the key findings for each data set were identified, discussed and agreed upon by consensus by SA, ST-C and KH. The key findings for each of the four data sets were presented as statements to aid comparison, e.g. “patients reported that the booklet was useful in providing information about their illness” [11]. Key findings represented each individual finding within each data set that was reported in the final report of each study. For the qualitative studies, multiple key findings were identified within each original theme, as themes were too broad in their descriptions to compare to quantitative findings. Once identified, key findings could be triangulated. The three researchers worked independently to triangulate the four data sets. Each key finding from one of the four data sets was compared to every other key finding in the other three data sets to create a “convergence coding matrix” [1]. The overall number of key findings reduced if the same findings were found in more than one data set. The matrix displayed the final list of key findings emerging from the four data sets on one page.

**Fig. 1:**
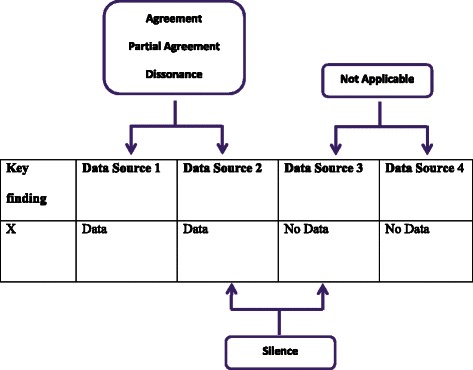
The five categories possible as a result of each pairwise comparison between data sets

For each key finding, paired comparisons were made to compare the data coming from each data set. The relationship between data was marked as one of four categories: silence, dissonance, partial agreement and agreement (Fig. 1). Agreement representing convergence in the data, partial agreement reflecting complementarity between data and dissonance reflecting conflicting findings in the data [1]. Silence reflected instances where only one data set out of the two being compared contained data on a particular finding. Comparisons were labelled as not applicable when neither data set in a paired comparison contained data related to the finding [1]. The three individual analyses, from each researcher, were then compared and discussed between the research team to obtain a consensus about the relationship between findings.

## Results

### Descriptive data

**Fig. 2:**
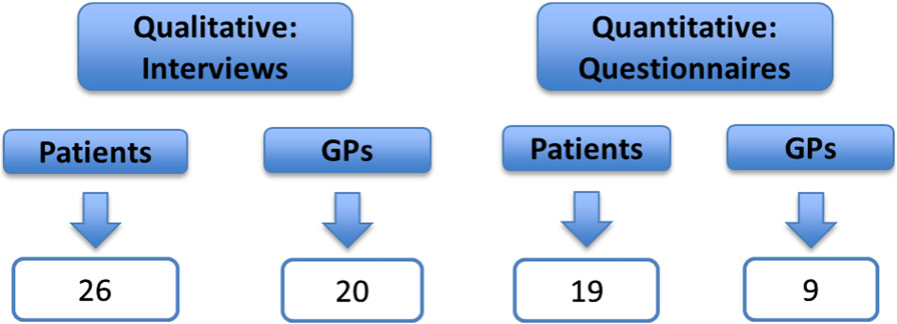
The 74 key findings identified in the original four data sets

Four data sets were collected. Qualitative data were collected from interviews with (1) 62 patients and (2) 66 GPs [6, 7]. Quantitative data consisted of self-report measures completed by (3) 2886 patients and (4) 346 GPs [8]. As a result of the mapping exercise, 74 independent key findings were identified in the four data sets (Fig. 2).

Three analysts compared all 74 findings to identify any overlap between data sets (where more than one data set found the same key finding) and to create a convergence coding matrix (see Additional file 1). Thirty-nine independent findings were identified across the four data sets. Triangulations carried out by individual investigators were compared to one another. All investigators agreed on the categorisation of 33/39 findings. The remaining 6 statements were discussed and agreed by consensus. Table 1 shows an example of one key finding identified.

**Table 1:** An example of one of the 39 key findings found across three of the data sets

Key finding	GP quantitative	Patient quantitative	GP qualitative	Patient qualitative
12	–	The usefulness of the booklet was rated as high by patients.	GPs said that the booklet addressed patients’ concerns and reinforced the explanation from the GP.	Patients reported that the booklet had new information about their illness which was valuable.

Thirteen findings related to a single data set, 11 findings appeared in two data sets and 15 findings appeared in three of the data sets. None of the key findings appeared in all four of the data sets. For each of the 39 statements, 6 comparisons were made to ensure completeness (Table 2). This led to a total of 234 paired comparisons across all data sets and key findings (Table 3). Of these, 58 were “not applicable” because there was no source data available for either data set; 123 were categorised as “silent” because there was no source data for one data set.

**Table 2:** The six pairwise comparisons between the four data sets for each key finding

Data set	GP quantitative	GP qualitative	Patient quantitative	Patient qualitative
GP Quant	X	1	2	3
GP Qual	1	X	4	5
Patient Quant	2	4	X	6
Patient Qual	3	5	6	X

**Table 3:** The number of statements within each convergence category resulting from pairwise comparisons

Convergence category	Number of statements
Agreement	18
Partial agreement	32
Dissonance	3
Silence	123
Not applicable	58
Total	234

### Interpretive analysis

#### Instances of dissonance

Statements which indicated dissonance between the data sets were of most interest. The first instance indicated dissonance between the two qualitative data sets. Patients appeared to have no preference between the patient booklet and the CRP test when used in consultations. Patients in intervention group 4, where both interventions were used, reported that they thought that both interventions were useful. Patients specifically reported that the CRP test was useful for the GP and perceived it as an additional clinical instrument which could help distinguish between a viral and bacterial illness. Patients felt that the test was for the GP only and many reported that they were confident in the GPs’ decision about a prescription regardless of whether or not the test had been done.

When GPs spoke about the interventions, they specified that the CRP test and patient booklet were suited to different groups of patients. GPs felt that the booklet could be used with patients who accepted a straightforward no-antibiotic decision. GPs felt that the CRP test was either for when there was diagnostic uncertainty or when patients expected antibiotics when they were not needed. In the latter context, GPs felt that having a test result, to back up their own explanation, helped to convince patients that a no-antibiotic decision was appropriate. Comparing the findings from the GP and patient data, GPs appeared to overestimate the need to use a CRP test to convince patients of a no-antibiotic decision.

The remaining two instances of dissonance were between the quantitative and qualitative data collected from patients. Two findings collected in the quantitative data identified that (1) patients in the CRP only intervention group and (2) those who had received a CRP test both reported lower satisfaction (These groups differed because not all the patients in the CRP only group, received the CRP test. Equally those who had received the test were either in the CRP only group or the combined intervention group). Whilst satisfaction with the consultation was generally high for all patients, it was lowest in those who had received a CRP test and highest in patients who had been in a communication group and received a booklet. When examining the data, it was apparent that different proportions of patients in the quantitative and qualitative samples had received a CRP test. Of those interviewed who could have had a test, 87 % had received one. In contrast, 38 % of all patients in the two intervention groups with CRP test had had a test carried out. This reflected the purposive sampling for the qualitative study which aimed to capture the views of patients who had had the test. The differences in patient satisfaction between different intervention groups may either reflect dissatisfaction with the CRP test, dissatisfaction when not receiving a test which patients know is available, or greater satisfaction with the communication groups and patient booklet. The qualitative data indicated that, for those individuals interviewed, there was minimal dissatisfaction with the CRP test and high satisfaction in all intervention groups; however, patients with more positive experiences may have been more likely to volunteer for interviews. The quantitative data, capturing a greater number of experiences, provides more information. Patients receiving a detailed explanation from their GP about their symptoms and receiving a booklet to take home with self-care advice likely increased the satisfaction in the communication groups. Questionnaire measures likely helped to quantify this difference in the trial population.

#### Instances of agreement

There were several instances of agreement between the data sets, and for many, qualitative data helped to explain findings in the quantitative data.

The quantitative data found that both intervention groups containing communication skills were perceived to help the reduction of prescribing and increase GP confidence in not prescribing. This was in comparison to the control group and CRP only group. Qualitative findings from the GP study helped to explain this. GPs reported that whilst CRP helped decrease diagnostic uncertainty, the test was only useful for a minority of consultations and that even when the test was used there were unaddressed issues. GPs reported that whilst the test helped to convince patients of a no-antibiotic decision, the test did not help to educate patients about the appropriate use of antibiotics in general or specifically address patient expectations for treatment of self-limiting infections. In addition, GPs reported that it was difficult to carry out the test in practice as it was complicated and took a significant amount of time in the consultation.

A second example was in the patient data. Patients answering the questionnaire who were (a) in the CRP only group and (b) those who had received a CRP test reported lower enablement to self-manage their illness than those who received the booklet. As mentioned above, these data sets were measuring different populations in terms of the proportions who had received a CRP test. In interviews, patients reported being happy with the booklet with many stating that they would use it to self-care and refer to it for future illness for themselves and their family. This indicated that patients in the communication groups felt more able to assess their symptoms and more informed about when to consult in the future. In contrast, a minority of patients who had received the CRP test reported that they would consult for the test in the future if they were ill, indicating that they felt more reliant on the doctor and the test.

#### Instances of silence

There were a significant majority of pairwise comparisons between data sets which resulted in silence. Silences were often between data sets reporting patient and GP opinions as data reported by one population was not relevant to the other. For example, patients reported on whether they felt antibiotics were necessary for cough; however, GPs did not comment directly on patient perceptions of the necessity of antibiotics. A number of comparisons were “not applicable” which occurred when two or more data sources provided no relevant data for a specific finding. This was often due to the retrospective nature of this study design and a result of questionnaire items and interview guides being developed separately by different researchers and thus having fewer areas of overlap.

## Discussion

### Main findings

The triangulation protocol added to and enhanced the findings of a process evaluation of a RCT of a complex intervention. The triangulation of data generated new findings and offered insights into the original findings from the previous analyses of data sets carried out individually. We found that following a triangulation protocol was a straightforward approach which could be carried out relatively easily by the same researchers who carried out the original analysis. A key new finding for the process evaluation of the trial was that GPs perceive that the CRP test is useful in persuading patients to agree to a non-antibiotic approach, but patient data suggested this was unnecessary if a full explanation about the disease and non-antibiotic management was given.

### Strengths and limitations

This study used a novel design by undertaking a triangulation protocol to analyse mixed methods data collected as part of a process evaluation of a trial of a complex intervention. A triangulation protocol is something which could be used in process evaluations of other RCTs to provide additional insights into how complex interventions could be implemented in practice. The analysis for this study benefitted from the large data sets collected for both the quantitative and qualitative data which captured a range of views.

Triangulation was achieved through using different data collection methods, different types of data and also by using multiple analysts. Despite these strengths, the study would have benefitted from being designed prospectively to allow the design of complementary interview guides and questionnaires. Individual patient and GP data could have been linked between quantitative and qualitative data sets; however, due to the number of participants involved, this was not feasible in the time available and is an approach which could be planned in a prospective design.

### Implications for research and practice

The triangulation protocol allowed new findings to emerge from the data which informed the process evaluation of the trial. GPs and patients appeared to have different views on the potential use of a CRP test. GPs’ desire to use tests to convince patients of a no-antibiotic decision seemed unnecessary when patients reported they were convinced by an explanation and/or patient booklet. These results are similar to previous research which indicates that perceived patient demand for antibiotics by the GP is greater than actual patient expectations for a prescription when there is diagnostic uncertainty [12, 13]. This finding helps to inform previous qualitative work which also found that GPs felt tests would convince patients of a no-antibiotic prescribing decision [14, 15]. Use of CRP tests is therefore only likely to be useful when there is diagnostic uncertainty and highlights research which suggests tests may only be useful for a subset of patients [16]. This reflects the inclusion of CRP tests in recent UK and Dutch guidelines on the management of suspected pneumonia [17]. Existing research corroborates the suitability of these interventions for primary care and indicates that training in communication skills can decrease antibiotic prescribing in the long term. In addition, both types of intervention appear to avoid any increase in reconsultation [18].

Assessing patient satisfaction with the CRP test was difficult. When dissonance arose, however, qualitative interviews may have resulted in some form of selection bias and likely captured patients who were satisfied with their consultation. The reports of the larger patient group, represented in the quantitative data set, are likely to reflect a more representative view. This finding suggests that patients in the communication groups were more satisfied with their consultations and this was likely a result of a detailed explanation by the GP and receiving a patient booklet for use at home. This is similar to previous research which indicates that GPs and patients are enthusiastic about written materials which give information about the illness [19–21]. This instance acts as an example to indicate how a single data set may provide a limited understanding of a particular phenomenon.

The triangulation of data between the four data sets provided a more holistic view of how communication skills and the CRP test were being used in practice within the trial. The CRP test did not help to educate patients or appear to increase patient satisfaction with the consultation and led to difficulties in practice by being difficult to use and taking up consultation time. In addition, GPs felt that the test was only useful for a minority of patients where there was clinical uncertainty with the risk that patients could return for the test in future. In contrast, the communication skills intervention was well received by patients and clinicians, helped to educate patients, supported clinician-patient interactions and increased patient satisfaction through acknowledging and addressing patient concerns. These results support the focus on shared decision-making initiatives in the context of acute respiratory infections as opposed to improved diagnostics [22].

Mixed methods research is becoming more prevalent in the medical literature, and there is a particular focus on how mixed methods can inform the process evaluations of complex interventions [23, 24]. Many studies of complex interventions may utilise both quantitative and qualitative aspects. However, the majority of these studies report quantitative and qualitative analyses separately and do not use methods to integrate findings [1, 25]. This is unlikely to be due to fundamental differences in the epistemological assumptions behind qualitative and quantitative research if studies are part of a larger work package but may instead reflect the practicalities of carrying out research [26]. Teams are likely to be formed of a number of methodologists and it may be more cost and time effective for each to work independently on individual data sets. This study provides an example of using a triangulation protocol to build on individual analyses to integrate mixed methods data in a cost effective and timely way. As other researchers have recognised, using mixed methods techniques in this way helps to produce an assessment which benefits from the strengths of each method whilst countering the limitations of each [27]. Use of a triangulation protocol in process evaluations of trials of complex interventions is likely to be highly valuable to researchers in determining how interventions can be used in practice.

## Conclusions

Use of CRP tests does not appear to engage patients or influence illness perceptions and its effect is more centred on changing clinician behaviour. Communication skills and the patient booklet were relevant and useful for all patients and associated with increased patient satisfaction. A triangulation protocol to integrate qualitative and quantitative data can reveal findings that need further interpretation and also highlight areas of dissonance that lead to a deeper insight than separate analyses.

## Additional file

Additional file 1: Convergence coding matrix. (XLSX 13 kb)
